# A chromosome-level genome assembly of the Asian giant softshell turtle *Pelochelys cantorii*

**DOI:** 10.1038/s41597-023-02667-1

**Published:** 2023-11-01

**Authors:** Xiaoyou Hong, Haiyang Liu, Yakun Wang, Mingzhi Li, Liqin Ji, Kaikuo Wang, Chengqing Wei, Wei Li, Chen Chen, Lingyun Yu, Xinping Zhu, Xiaoli Liu

**Affiliations:** 1 Key Laboratory of Tropical and Subtropical Fishery Resources Application and Cultivation, Ministry of Agriculture and Rural Affairs, Pearl River Fisheries Research Institute, Chinese Academy of Fishery Sciences, Guangzhou, 510380 China; 2 Guangzhou Bio&data Technology Co., Ltd, Guangzhou, 510555 China; 3 College of Life Science and Fisheries, Shanghai Ocean University, Shanghai, 201306 China

**Keywords:** Genomics, Genome

## Abstract

The Asian giant softshell turtle *Pelochelys cantorii* is one of the largest aquatic turtles in China and has been designated a First Grade Protected Animal in China. To advance conservation research, a combination of Illumina short-read, PacBio long-read, and Hi-C scaffolding technologies was used to develop a high-quality chromosome-level genome assembly for *P. cantorii*. A total of 262.77 Gb of clean data were produced (121.6 × depth) and then the genome was assembled into 2.16 Gb with a contig N50 of 41.44 Mb and scaffold N50 length of 120.17 Mb, respectively. Moreover, about 99.98% assembly genome sequences were clustered and ordered onto 33 pseudochromosomes. Genome annotation revealed that 21,833 protein-coding genes were predicted, and 96.40% of them were annotated. This new chromosome-level assembly will be an enabling resource for genetic and genomic studies to support fundamental insight into *P. cantorii* biology.

## Background & Summary

The Asian giant softshell turtle *Pelochelys cantorii* is one of the largest aquatic turtles and is widely distributed in Southeast Asia. With rapid economic development, the numbers of *P. cantorii* have sharply declined due to overhunting and habitat destruction in China. Although listed as a national first-class protected animal in China in 1989, wild *P. cantorii* individuals in China have been reported at a declining rate in the past 30 years^1,2^, and only 13 *P. cantorii* individuals from the wild are in captivity across 6 different locations. *P. cantorii* is extremely endangered in China^1,3^. The fate of another large soft-shelled turtle in China, the Yangtze giant softshell turtle, *Rafetus swinhoei*, is even more worrisome. No individuals were found in the wild, and two individuals (1 female and 1 male) reared in captivity failed in assisted reproduction after 2008. In 2019, the lone female *R. swinhoei* died during artificial insemination. If there are no *R. swinhoei* individuals in the wild, the fate of *R. swinhoei* is sealed. If the protection of *P. cantorii* is not strengthened, it is inevitable that the *P. cantorii* will not become the next *R. swinhoei*.

In 2014, 10 cantorii hatchlings were successfully bred in captivity from 2 turtle individuals (1 female and 1 male)^4^. From 2015 to 2021, four captive *P. cantorii* individuals (2 females and 2 males) successfully bred and raised 950 juveniles that are currently 1–7 years old. Knowledge of the artificial breeding of *P. cantorii* offers hope for the conservation of this species^5^. With the breakthrough of the artificial breeding of *P. cantorii*, related protection work has received attention from the government of China. *P. cantorii* is listed as one of the nine aquatic wild animals under the key management of the Ministry of Agriculture and Rural Affairs (MARA) of the People’s Republic of China. In 2019, the MARA of China issued the “*Pelochelys cantorii* Rescue Action Plan (2019–2035)”. In September 2020, the MARA of China organized and carried out the first wild adaptation protection test for *P. cantorii*. A total of 20 juvenile turtles with implanted PIT chips were released in a reservoir in Gaoming, Foshan. These turtles were between 4–5 years old and weigh 1.04–1.66 kg.

With the development of sequencing technology, conservation genomics was born, which overcomes the limitations of conservation genetics, such as the lack of markers to a large extent, and helps to solve some unresolved problems in the conservation biology of organisms, especially endangered species^6^. For example, *de Novo* sequencing and population resequencing analysis based on Yangtze finless porpoise *Neophocaena Asiaeorientalis asiaeorientalis* more accurately determined the phylogenetic relationships and population genetic structure, and confirmed the independent species status of the Yangtze finless porpoise^7^. Through the analysis of genomic data of hot spring snakes, the genes involved in DNA damage repair (FEN1) and hypoxia response (EPAS1) were identified, providing research ideas for the mechanism of environmental adaptation. With the continuous development and optimization of high-throughput sequencing technology, research on conservation genomes will continue to improve.

Third-generation sequencing technologies, such as Pacific Biosciences (PacBio) Single Molecule Real-Time (SMRT) and Oxford Nanopore sequencing, characterized by single-molecule sequencing, can overcome the shortcomings of second-generation sequencing methods^8^. Hi-C, based on the combination of chromatin conformation capture and high-throughput sequencing, reveals the interaction information between chromosome fragments through the analysis of sequencing data^9–12^.

Tortoises are an ancient reptile with more than 300 species and have significant evolutionary and ecological value. No more than 10 turtle genome projects have been completed since the first investigation on turtle genomes were published in 2013 (Chinese soft-shell turtle and green turtle). Therefore, it is urgent to expand the diversity of the turtle genome databases.

Here, we sequenced and *de novo* assembled the genome of a young *P. cantorii* via a combination of long-read PacBio Sequel II platform and Hi-C sequencing technology. The high-quality reference genome constructed in this study will not only be of benefit to serve as the genetic basis for in-depth investigations of turtle evolution and biology but also offers a valuable genetic resource for turtle conservation.

## Methods

### Ethics statement

All the experimental procedures regarding the turtle involved in this experiment were approved by the Experimental Animal Care and Ethics Committee of the Pearl River Fisheries Research Institute, Chinese Academy of Fishery Sciences.

### Sample preparation and genome sequencing

**Fig. 1:**
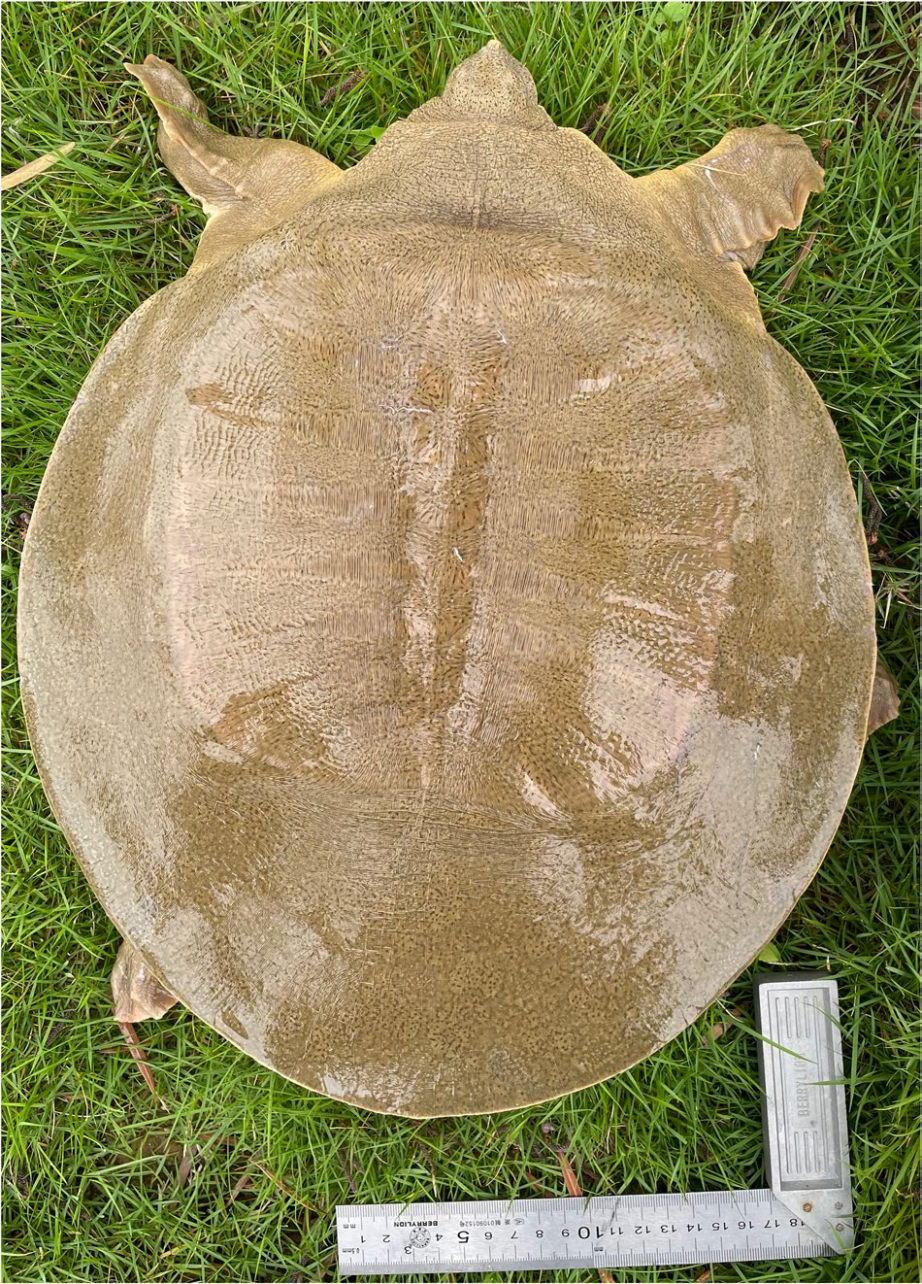
Artificially bred 5-year-old Asian giant softshell turtle.

A healthy 2-year-old adult was obtained from the breeding center of the Pearl River Fisheries Research Institute, Guangzhou, Guangdong, China (Fig. 1). The turtle was anaesthetized using tricaine methanesulfonate (MS-222, Sigma) before sampling. The fresh muscle tissue was cut to small sizes (about 20 µg) and conducted for DNA extraction using a DNeasy Blood & Tissue Kit (Qiagen, Valencia, CA, USA), according to the manufacturer’s instructions. Only high-quality DNA can be used for the following library preparation, sequencing, and Hi-C library construction, so the quantity and quality of the genomic DNA were first evaluated by using Qubit 3.0 (Life Invitrogen, USA) and 1% agarose gel electrophoresis, respectively. The library was then constructed and sequenced on Illumina HiSeq4000 (Illumina, San Diego, CA, USA) and the PacBio Sequel II platform according to the manufacturer’s instructions.

Tissues from the heart, liver, kidney, muscle, eye, blood, and lung of the same turtle were used for RNA extraction by TRIzol Universal Reagent (TIANGEN Biotech, Beijing, China). Two micrograms of total RNA from each tissue were pooled for RNA sequencing on a PacBio Sequel II.

### Genome size estimation and initial genome assembly

**Fig. 2:**
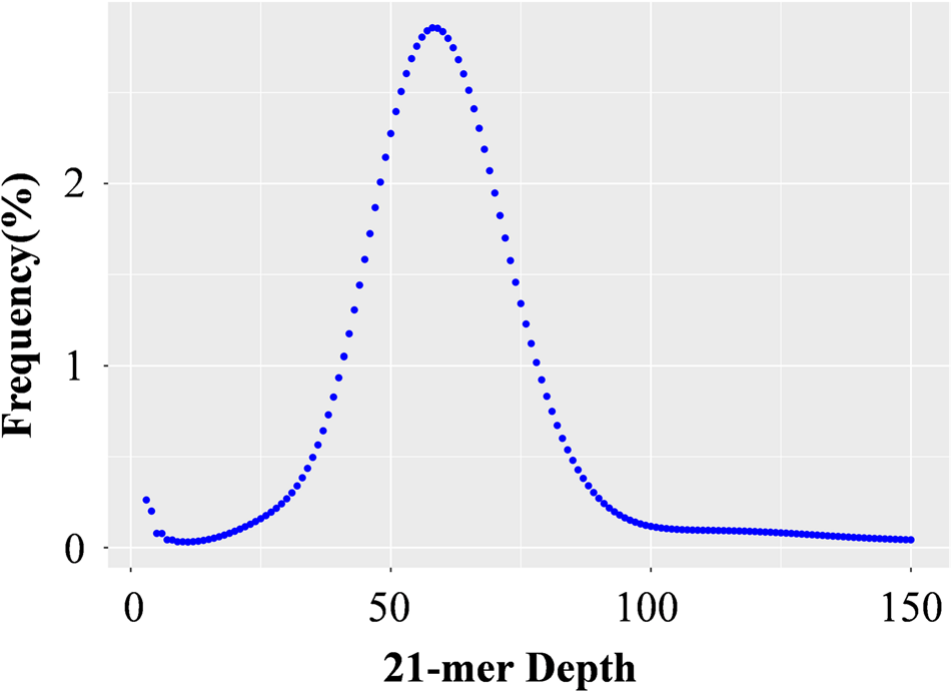
A 21-mer distribution of the Illumina short reads to estimate genome size, ratio of repeat sequences and heterogeneity. The x-axis represents the sequencing depth of each unique 21-mer, and the y-axis represents the frequency of unique 21-mers. The k-mer depth value of the main peak is 56. After removing data with abnormal depth, a total of 131,112,165,086, 21-mers were used to further estimate the genome size; thus, we first estimated that the genome size of the Asian giant softshell turtle was 2.20 Gb.

Genome size was estimated by *k*-mer analysis^13^ using filtered reads from the three 350 bp libraries constructed using genomic DNA and a *k*-mer size of 21. Genome size, repetition ratio, and heterozygosity were assessed by *k*-mer depth frequency distribution analysis (Table 1). The average *k*-mer depth was 56, and the total number of *k*-mers obtained from sequencing data was 131,112,165,086, further estimating the genome size of *P. cantorii* (Fig. 2 and Table 1). Sequences with a *k*-mer depth greater than 113 were repeated sequences, and those with a *k*-mer depth of approximately 28 were heterozygous sequences. After removing *k*-mers of abnormal depth, the genome size of *P. cantorii* was evaluated to be 2.20 Gb (based on the following formula: G = *k*-mer number/mean *k*-mer depth), with a low heterozygosity of approximately 0.14% and a repeat sequence content of 26.29% (Table 1). The PacBio sequencing platform generated a total of 262.77 Gb of clean data (approximately 121.60 × ), with a read N50 of 22.85 kb and an average read length of 14.62 kb (Table 2). The data were first assembled with WTDBG2, which generated a genome assembly with a total length of 2.16 Gb, 548 contigs, and 41.44 Mb contig N50 (Table 3).

**Table 1:** The data statistic of k-mer analysis and heterozygosity in the Asian giant softshell turtle.

*k*-mer	*k*-mer depth	*k*-mer number	filtered *k*-mer number	Genome size (Gb)	Heterozygosity (%)	Repeat sequence content (%)
21	56	131,112,165,086	125,237,543,723	2.20	0.14	26.29

**Table 2:** Statistics of short-reads illumina in the Asian giant softshell turtle genomic survey.

Reads Type	Reads Num	Total Bases(bp)	Reads N50 (bp)	Reads mean Length(bp)	Longest Read (bp)
Subreads	17,978,047	262,768,273,418	22,851	14,616	283,173
ZMWreads	13,948,886	199,616,601,708	22,488	14,311	283,173

**Table 3:** Statistics of the genome assembly, Hi-C results and gene sent.

Parameter	Scaffold	Contig
**Genome assembly and Hi-C result**		
Total No.	65	555
Average length (bp)	2,161,043,548	2,160,994,548
N50 length (bp)	120,169,148	41,440,143
N90 length (bp)	27,391,981	7,000,000
Maximum length (bp)	348,738,502	181,665,310
GC content (%)	45.30	45.30
**Gene annotation**		
Protein-coding gene No.	—	21,833
Mean Gene length (bp)	—	37,890.68
Mean exons per gene	—	9.38
Mean exon length (bp)	—	2,428.28
Mean intro length (bp)	—	35,462.40

### Chromosome assembly using Hi-C data

A high-throughput chromatin conformation capture (Hi-C) library was then created for sequencing from 2-year-old individual muscle tissue. The sample was fixed with paraformaldehyde and enzymatically digested with DnpII, generating sticky ends. The sticky ends can be repaired by ‘A’ or ‘C’ deoxynucleotides with biotin under the action of DNA polymerase. After labeling the 5′ overhang with a biotinylated residue, the DNA fragments were ligated to each other to form chimeric circles using DNA ligase. Then, the ligated DNAs were uncrosslinked, purified, and sheared to 300 bp-700 bp fragments, followed by capture with streptavidin beads and preparation for sequencing. Finally, the Hi-C libraries were quantified and sequenced using the Illumina HISeq X 10 platform in PE150 mode with paired-end methods.

To obtain high-quality Hi-C data, the joint information, low-quality bases, and undetected bases in the original sequencing data were first removed to generate clean reads. Second, BWA v0.7.10-r789 was applied to align the clean reads to the draft genome assembly with the default parameters. HiC-Pro v2.8.1 was performed to filter invalid read pairs, including dangling-end and self-cycle, re-ligation, and dumped products. The contigs of the primary genome assembly were corrected by splitting them into segments of 50 kb on average. After checking any two segments that showed inconsistent connection with information from the raw scaffold, LACHESIS (ligating adjacent chromatin enables scaffolding *in situ*) was used to produce chromosome-level scaffolds. The final assembled genome was 2.16 Gb and anchored 532 contigs. The scaffold N50 was 41.44 Mb with a fixed rate of 95.86% (Table 3). Among the sequences located on chromosomes, the length of the sequence that can determine the order and direction is up to 99.97%. The Hi-C scaffolding of the genome resulted in 33 chromosomes (Chr) (Figure S1), accounting for 99.98% of the total assembly (Table 4). In order to further determine the chromosome number of the Asian giant softshell turtle, about 1 mL blood was extracted from the neck blood vessels of four 1 to 3 years-old young individuals from the turtle domestication base of the Pearl River Fisheries Research Institute. After 72 h of culture, cells were collected for the preparation of chromosomes analysis as previously described^14^. A total of 43 metaphase mitotic cells with a higher number of chromosomes were obtained (Figure S2, Figure S3). Finally, we selected one of the chromosome division phases to make the standard karyotype map (Figure S4), and the karyotype formula was determined by the first eight division phase maps in Supplementary figure 1.

**Table 4:** Table summary of the assembled chromosomes of the Asian giant softshell turtle.

Chr	Number of Contigs	Length of Contigs (bp)	Number of Contigs Num determine order	Length of Contigs (bp)
Chr01	20	348,736,602	20	348,736,602
Chr02	21	264,231,402	21	264,231,402
Chr03	9	193,892,150	9	193,892,150
Chr04	7	133,002,195	7	133,002,195
Chr05	13	132,210,151	13	132,210,151
Chr06	6	120,168,648	6	120,168,648
Chr07	8	76,745,611	8	76,745,611
Chr08	8	75,773,209	8	75,773,209
Chr09	18	68,048,706	18	68,048,706
Chr10	7	57,045,259	6	57,020,171
Chr11	4	54,320,897	4	54,320,897
Chr12	1	49,949,040	1	49,949,040
Chr13	5	48,483,669	5	48,483,669
Chr14	9	47,304,674	9	47,304,674
Chr15	2	43,234,547	2	43,234,547
Chr16	5	42,474,419	5	42,474,419
Chr17	13	39,727,757	5	39,093,329
Chr18	2	37,160,797	2	37,160,797
Chr19	2	31,099,951	2	31,099,951
Chr20	217	28,105,451	217	28,105,451
Chr21	3	28,055,190	3	28,055,190
Chr22	1	27,391,981	1	27,391,981
Chr23	4	26,063,040	4	26,063,040
Chr24	9	24,214,670	9	24,214,670
Chr25	6	21,149,485	6	21,149,485
Chr26	109	21,073,363	109	21,073,363
Chr27	5	20,515,901	5	20,515,901
Chr28	7	19,492,991	7	19,492,991
Chr29	3	18,069,597	3	18,069,597
Chr30	4	17,638,359	4	17,638,359
Chr31	1	16,634,628	1	16,634,628
Chr32	1	15,000,000	1	15,000,000
Chr33	2	13,643,742	2	13,643,742
Total (Ratio %)	532(95.86)	2160658082 (99.98)	523 (98.31)	2159998566 (99.97)

**Fig. 3:**
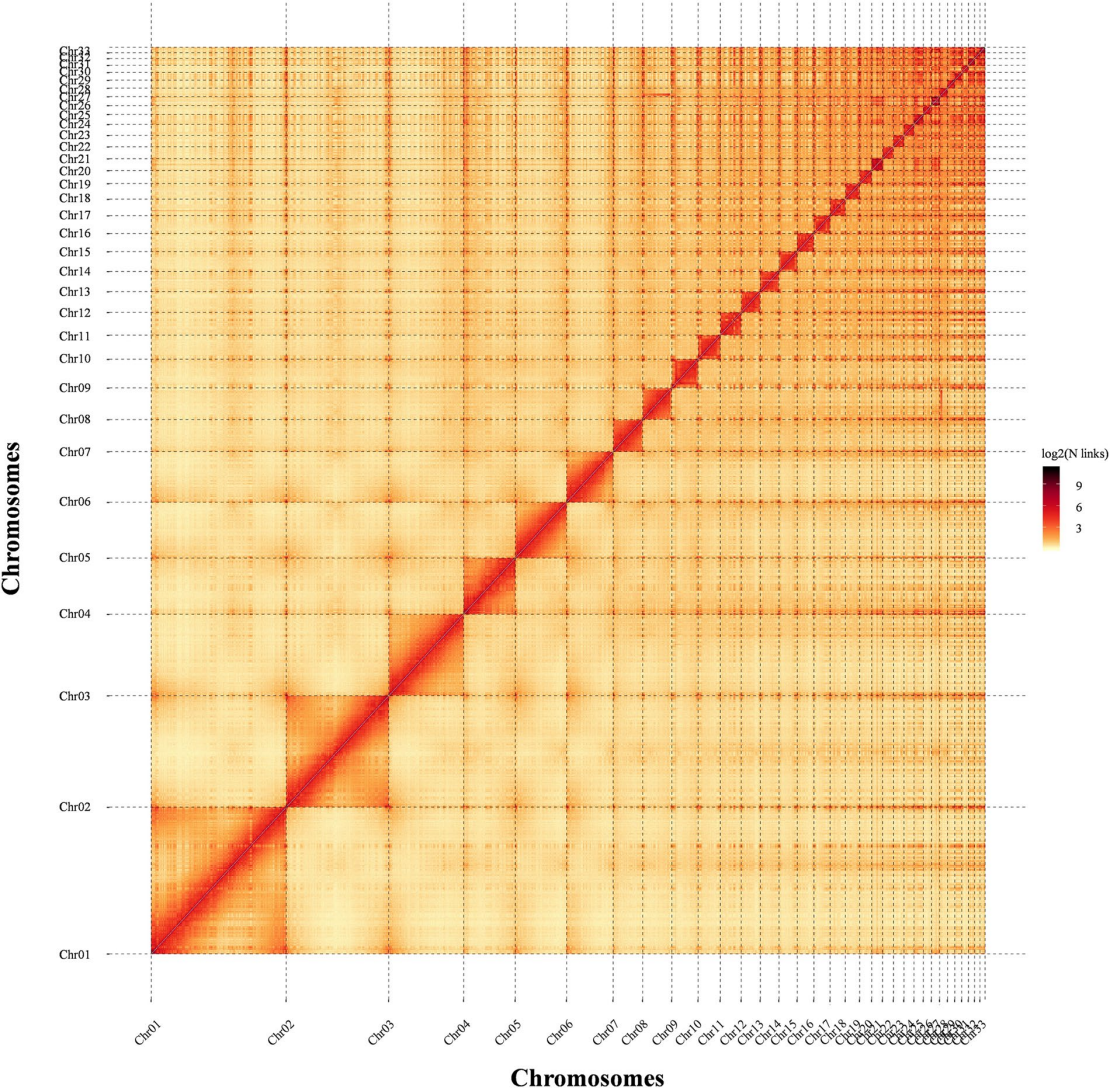
Hi-C assembly of Chromosomal interaction maps. The 33 squares represent the constructed 33 chromosomes (Chr01 – Chr33) of the Asian giant softshell turtle. The color from light (low) to dark (high) indicates contact density of Hi-C interaction links.

Subsequently, Juicer and JucieBox v.1.8.8 were implemented to construct an interactive map and correct assembly errors visually, respectively. The heat map of the Hi-C assembly interaction bins displayed stronger signals around the diagonal than that of other positions. The phenomenon revealed that intensity of the interaction between adjacent sequences (diagonal position) was high, while the intensity of the interaction signal between non-adjacent sequences (non-diagonal position) was weak, which was consistent with the principle of Hi-C assisted genome assembly, proving a high quality and completeness of a genome assembly (Fig. 3).

### Assessment of the genome assemblies

**Fig. 4:**
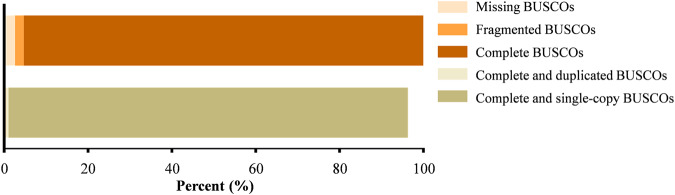
Genome completeness of the Asian giant softshell turtle genomic sequence evaluated by BUSCO v3.0. The colours represent different kind of BUSCOs the Complete BUSCOs, Fragmented BUSCOs, Missing BUSCOs, Complete and duplicated BUSCOs and Complete and single-copy BUSCOs.

Two methods were used to check the completeness and quality of the assembly. First, BWA v0.7.10-r789^15^ was used to compare the short sequences obtained by second-generation high-throughput Illumina sequencing with the reference genome, and the integrity of the assembled genome was evaluated by the alignment rate. Approximately 98.95% of clean reads were mapped to contigs, and 96.96% of clean reads were mapped to proper pairs (Table 5). Second, BUSCO v3.0^16^ was used to search the 2,586 universal single-copy orthologous genes in vertebrata_OrthoDB v9 to evaluate the completeness, degree of fragmentation and missing genes of the genome assembly. Among the 2,586 prospective conserved core genes in the eukaryotic database, 2,478 (95.82%) and 54 (2.09%) were identified as complete and fragmented BUSCOs, respectively, indicating that the assembled genome had high integrity and validity, and could be used for further analysis (Fig. 4, Table 6).

**Table 5:** The alignment of the Illumina reads to the Asian giant softshell turtle genome assembly.

Total_reads	Mapped_reads	Mapped(%)	Properly_mapped_reads	Properly_mapped(%)
1,018,142,445	1,007,498,266	98.95	981,840,892	96.96

**Table 6:** Quality assessment of genome using vertebrata_ OrthoDB v10 database.

Term	BUSCO number	Proportion(%)
Complete BUSCOs	2,478	95.82
Complete and single-copy BUSCOs	2	
464	95.28	
Complete and duplicated BUSCOs	14	0.54
Fragmented BUSCOs	54	2.09
Missing BUSCOs	54	2.09
Total BUSCO groups searched	2,586	100.00

### Gene prediction and functional annotation

With the help of LTR_FINDER^17^ and RepeatScout^18^, a repeat sequence database of the genome was constructed, based on the principle of structure prediction and *ab initio* prediction. PASTEClassifier^19^ was used to categorize different types of repetitive sequences. The ultimate repeat library was subsequently determined combined with Repbase^20^ database. At last, RepeatMasker^21^ software was conducted to predict the repeated sequences. We identified 993,573,618 bp sequences as repeats in the Repbase database, which covered 45.98% of the genome assembly (Table 7). The most common repetitive elements, RNA transposons (class I), account for 39.33% of the genome content, higher than DNA transposons (Class II) (Table 7). Long interspersed nuclear elements (LINEs) were the most abundant repeating element, followed by large retrotransposon derivatives (LARDs) and terminal inverted repeats (TIRs) (Table 7). In addition, several sequences classified as unknown repeats accounted for 2.48% of the genome assembly (Table 7).

**Table 7:** The detailed repetitive elements in the genome.

Type	Number	Length(bp)	Rate(%)
**ClassI**	4,336,455	849,960,593	39.33
ClassI/DIRS	195,999	69,660,382	3.22
ClassI/LARD	1,308,272	249,747,120	11.56
ClassI/LINE	1,706,636	416,261,004	19.26
ClassI/LTR/Copia	2,472	316,980	0.01
ClassI/LTR/Gypsy	218,444	71,392,354	3.30
ClassI/LTR/Unknown	76,570	28,881,350	1.34
ClassI/PLE	813,859	189,340,452	8.76
ClassI/SINE	11,040	1,726,075	0.08
ClassI/TRIM	2,411	671,971	0.03
ClassI/Unknown	752	97,976	0.00
**ClassII**	886,516	207,678,021	9.61
ClassII/Crypton	474	29,960	0.00
ClassII/Helitron	5,087	543,116	0.03
ClassII/MITE	48,753	11,607,987	0.54
ClassII/Maverick	5,558	1,356,319	0.06
ClassII/TIR	803,189	193,576,676	8.96
ClassII/Unknown	23,455	2,007,591	0.09
**PotentialHostGene**	9,085	1,674,363	0.08
**SSR**	355	62,966	0.00
**Unknown**	294,771	53,639,334	2.48
**Total**	5,527,182	993,573,618	45.98

A combination of three different strategies including *ab initio* prediction, homologous species prediction and transcriptome-based prediction were conducted to establish gene models. For gene predication based on *ab initio*, Genscan^22^, Augustus v2.4^23^, GlimmerHMM v3.0.4^24^, GeneID v1.4^25^, and SNAP version 2006-07-28^26^ were employed to predict protein-coding genes. For homology-based prediction, GeMoMa v1.3.1^27,28^ was used to align the assembled genome of *P. cantorii* with homologous species to predict the potential gene structures. Regarding RNA-Seq based prediction, Hisat v2.0.4^29^ and Stringtie v1.2.3^30^ were used for transcriptome assembly based on reference transcripts, and TransDecoder v2.0 and GeneMark-ST v5.1^31^ were used for gene prediction. PASA v2.0.2^32^ was used to predict Unigene sequences based on unreferenced assembly of transcriptome data. Finally, EVM v1.1.1^33^ software was used to integrate the prediction results of the above three methods, and PASA v2.0.2 was used to modify the results (Table 8).

**Table 8:** Statistics of gene prediction results.

Method	Software	Species	Gene number
*Ab initio*	Genscan	—	35,524
	Augustus	—	26,825
	GlimmerHMM	—	138,228
	GeneID	—	23,846
	SNAP	—	79,064
Homology-based	GeMoMa	*Platysternon megacephalum*	20,343
		*Pelodiscus sinensis*	21,068
		*Chrysemys picta*	20,353
		*Chelonia mydas*	19,591
		*Trachemys scripta*	19,061
		*Gopherus agassizii*	21,689
RNAseq	TransDecoder	—	67,822
	GeneMark-ST	—	35,316
	PASA	—	16,002
Integration	EVM	—	21,833

The predicted gene sequences were compared using BLAST V2.2.31^34^ (-evalue1e-5) with functional databases such as NR^35^, KOG^36^, GO^37^, KEGG^38^ and TrEMBL^39^. Gene functional annotation analysis, including KEGG pathway annotation analysis, KOG functional annotation analysis and GO functional annotation analysis, was performed. Finally, 21,833 protein-coding genes were successfully annotated. Of these predicted genes, 21,046 (~96.40%) were functionally annotated in at least one database, including GO, KEGG, KOG, TrEMBL and NR database (Table 9). Moreover, Blastn was used in the Rfam^40^ database for whole-genome comparison to identify microRNAs and rRNAs. tRNAscan-SE^41^ was used to identify tRNA. A total of 1595 tRNAs, 220 microRNAs and 86rRNAs were identified.

**Table 9:** Functional annotation from the genome assembly of the Asian giant softshell turtle.

Database	Annotated number	% of gene
GO_Annotation	7,103	32.53
KEGG_Annotation	13,401	61.38
KOG_Annotation	13,445	61.58
TrEMBL_Annotation	20,802	95.28
NR_Annotation	21,030	96.32
All_Annotated	21,046	96.40

## Data Records

The sequencing data have been deposited in the NCBI Sequence Read Archive database under the BioSample accession numbers. The accession number of coding sequences, Illumina, genome raw data, assembled genome data, gene annotation, exon annotation, Hi-C and full-length transcriptome sequences were SRR22715189^42^ (PRJNA910848), SRR22681424-SRR22681426^43^ (PRJNA911015), SRR22296394^44^ (PRJNA901634), SRR24179425^45^ (PRJNA955900), SRR22715197^46^ (PRJNA910853) and SRR22674657^47^ (PRJNA910849), SRR23047442^48^ (PRJNA922717) and SRR23047393^49^ (PRJNA922721) respectively. The genome assembly is available for public access at the NCBI GenBank under the accession number GCA_032595735.1^50^. Genome annotations (.gff3), along with predicted coding sequences (.cds) and protein sequences (.pep), can be accessed through the Figshare^51^. Moreover, all the data were also stored in zenodo database^52^.

## Technical Validation

Genomic integrity, fragmentation, and possible loss rates were measured using BUSCO V3. Among 2,586 prospective conserved core genes in the eukaryotic database, 2,478 (95.82%) and 54 (2.09%) were identified as complete BUSCOs and fragment BUSCOs, respectively, indicating that the assembled genome had high integrity and validity and could be used for further analysis (Fig. 4, Table 6).

### Supplementary information

Supplementary information

## Data Availability

All commands and pipelines used in data processing were executed according to the manual and protocols of the corresponding bioinformatics software. The settings and parameters of these softwares are described below. (1) BWA v0.7.10-r789: aln, default parameters; (2) LACHESIS: CLUSTER_MIN_RE_SITES = 547; CLUSTER_MAX_LINK_DENSITY = 2; CLUSTER_NONINFORMATIVE_RATIO = 2; ORDER_MIN_N_RES_IN_TRUN = 1094; ORDER_MIN_N_RES_IN_SHREDS = 1076; (3) BUSCO v3.0: --evalue 1e-03 (E-value cutoff for BLAST searches), -sp human; (4) LTR_FINDER: default parameters; (5) RepeatScout: default parameters; (6) PASTEClassifier: default parameters; (7) RepeatMasker: -nolow -no_is -norna -engine wublast; (8) Genscan: default parameters; (9) Augustus v2.4: default parameters; (10) GlimmerHMM v3.0.4: default parameters; (11) GeneID v1.4: default parameters; (12) SNAP: version 2006-07-28, default parameters; (13) GeMoMa v1.3.1: default parameters; (14) Hisat v2.0.4: --max-intronlen 20000, --min-intronlen 20; (15) Stringtie v1.2.3: default parameters; (16) TransDecoder v2.0: default parameters; (17) GeneMark-ST v5.1: default parameters; (18) PASA v2.0.2: -align_tools gmap, -maxIntronLen 20000; (19) EVM v1.1.1: default parameters; (20) BLAST V2.2.31: -evalue 1e-5; (21) tRNAscan-SE: default parameters.
