# Supplementary material for: A chromosome-level genome assembly of the Asian giant softshell turtle Pelochelys cantorii
Source: Sci Data. 2023 Nov 1;10:754. doi: 10.1038/s41597-023-02667-1 (PMC10620421; doi:10.1038/s41597-023-02667-1)
Supplement: Supplementary file 1 — Supplementary information [file 41597_2023_2667_MOESM1_ESM.docx]

**A chromosome-level genome assembly of the Asian giant softshell turtle** ***Pelochelys cantorii***

Xiaoyou Hong^1^, Haiyang Liu^1^, Yakun Wang^1^, Mingzhi Li^3^, Liqin Ji^1^, Wang Kaikuo^1,2^, Chengqing Wei^1^, Wei Li^1^, Chen Chen^1^, Lingyun Yu^1^, Xinping Zhu^1,2*^, Xiaoli Liu^1*^

1Key Laboratory of Tropical and Subtropical Fishery Resources Application and Cultivation, Ministry of Agriculture and Rural Affairs, Pearl River Fisheries Research Institute, Chinese Academy of Fishery Sciences, Guangzhou, 51038

2. College of Life Science and Fisheries, Shanghai Ocean University, Shanghai, China, 201306

3. Guangzhou Bio&data Technology Co., Ltd, Guangzhou, China, 510555


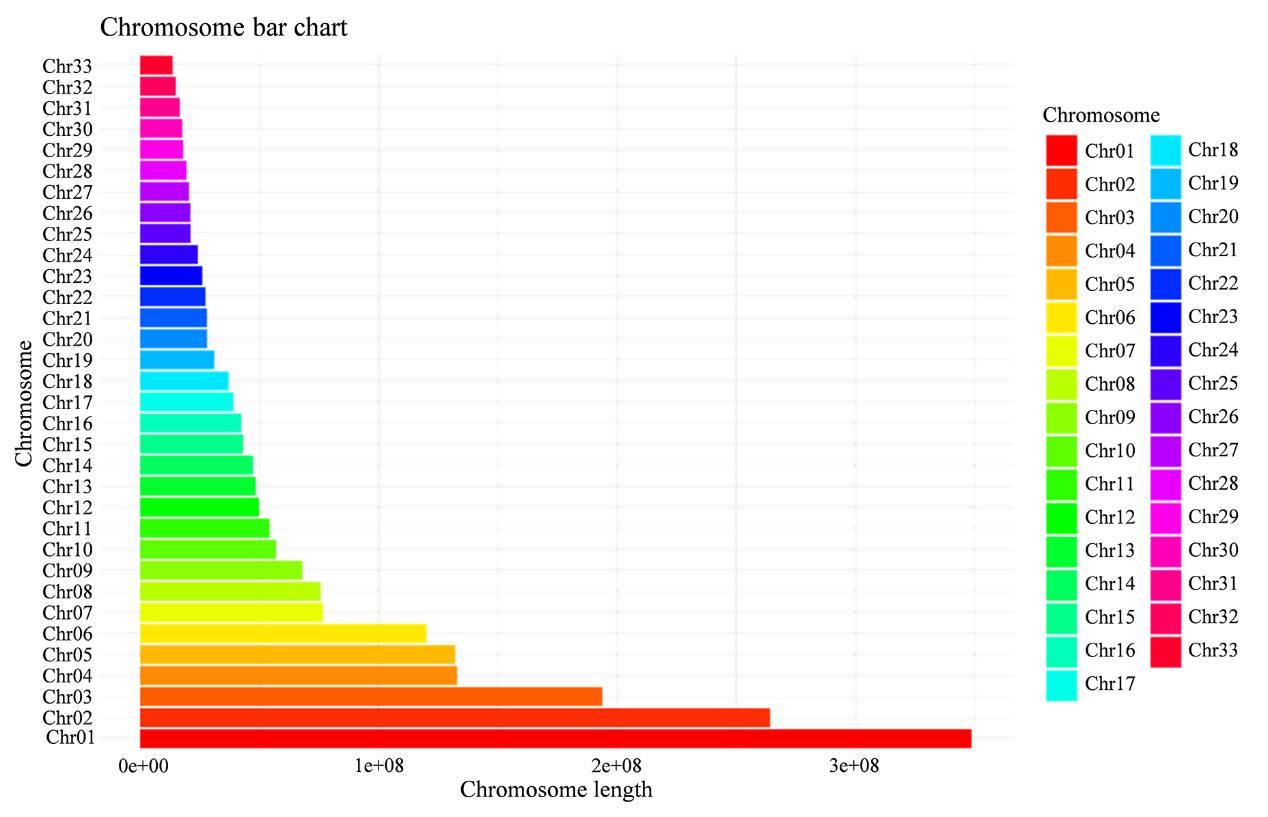


Figure S1. Chromosome bar chart of the Asian giant softshell turtle. “Chr” is an abbreviation for chromosome.


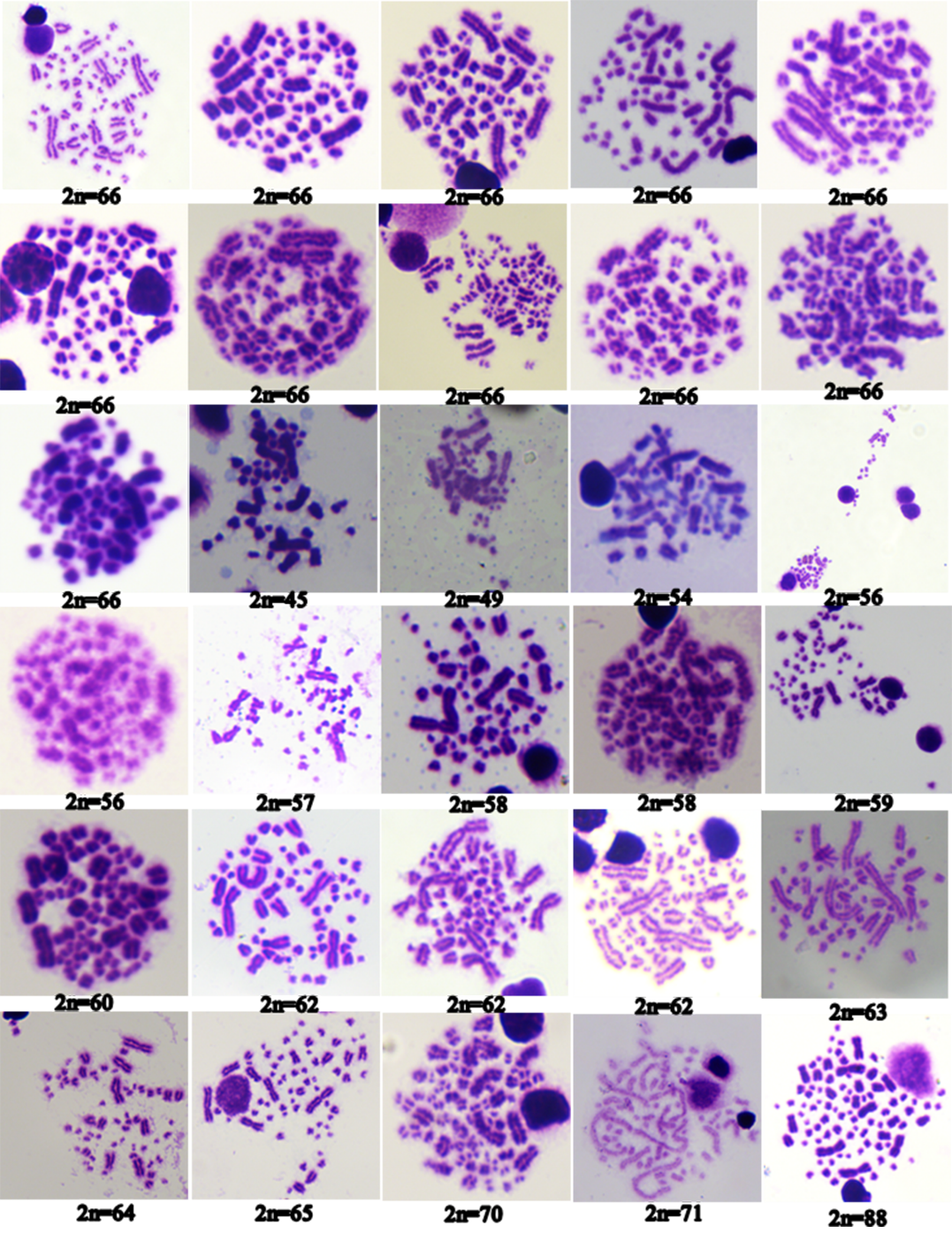


Figure S2. Mitotic phase and chromosome number of the Asian giant softshell turtle (Partial).


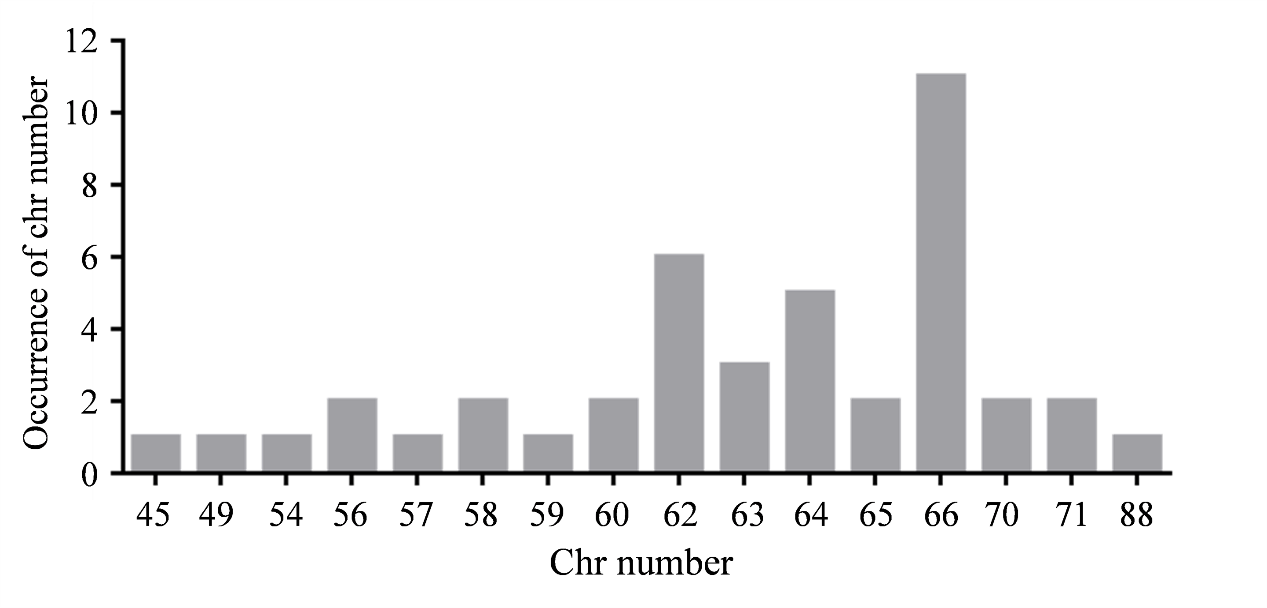


Figure S3. Number and frequency of metaphase mitotic phase of the Asian giant softshell turtle chromosome. The abscissa represents the number of turtle chromosomes and the ordinate represents the number of occurrences of that chromosome. “Chr” is the abbreviation for chromosome.


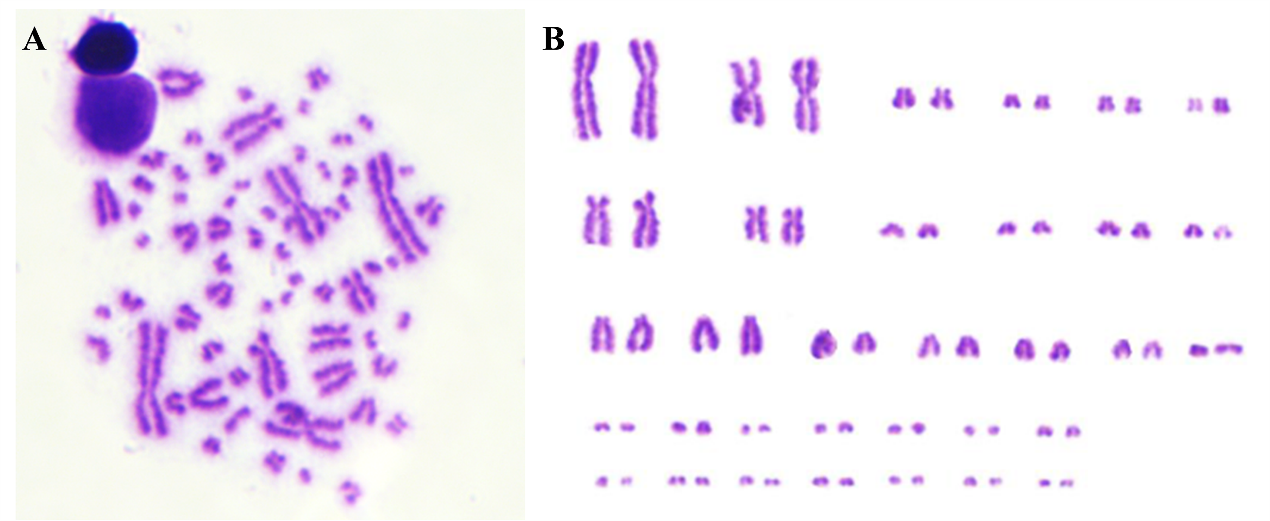


Figure S4. Chromosome mitotic phase and karyotype map of the Asian giant softshell turtle.
